# Associations of prenatal exposure to mixtures of organochlorine pesticides and smoking and drinking behaviors in adolescence

**DOI:** 10.1016/j.envres.2021.112431

**Published:** 2021-11-27

**Authors:** Aisha S. Dickerson, Zhengyi Deng, Yusuf Ransome, Pam Factor-Litvak, Oskar Karlsson

**Affiliations:** a Department of Epidemiology, Johns Hopkins Bloomberg School of Public Health, Baltimore, MD, USA, 615 N Wolfe Street, E7638, Baltimore, MD 21205, USA; b Department of Social and Behavioral Sciences, Yale School of Public Health, 60 College Street, LEPH 4th Floor, New Haven, CT 06510, USA; c Department of Epidemiology, Mailman School of Public Health, Columbia University, New York, NY 10032, USA; d Science for Life Laboratory, Department of Environmental Sciences and Analytical Chemistry, Stockholm University, Stockholm, 114 18, Sweden

**Keywords:** Alcohol, Smoking, In utero, DDT, DDE, OC pesticides, Persistent organic pollutants, Adolescent behaviors, Chemical mixtures

## Abstract

It is important to identify the factors that influence the prevalence of disinhibitory behaviors, as tobacco and alcohol use in adolescence is a strong predictor of continued use and substance abuse into adulthood. Organochlorine pesticides (OCPs) are persistent organic pollutants that pose a potential risk to the developing fetus and offspring long-term health. We examined associations between prenatal exposure OCPs and their metabolites (i. e., *p,p’*-DDT, *p,p’*-DDE, *o,p’*-DDT, oxychlordane, and hexachlorobenzene (HCB)), both as a mixture and single compounds, and alcohol consumption and smoking at adolescence in a sample (n = 554) from the Child Health and Development Studies prospective birth cohort. Bayesian Kernel Machine Regression demonstrated a trend of higher risk of alcohol use and smoking with higher quartile mixture levels. Single-component analysis showed increased odds of smoking and drinking with increases in lipid-adjusted *p,p*’-DDE serum levels (aOR = 2.06, 95% CI 0.99–4.31, *p* = 0.05, per natural log unit increase). We found significant effect modification in these associations by sex with higher *p,p’*-DDT serum levels (aOR = 0.26, 95% CI 0.09–0.076, *p* = 0.01, per natural log unit increase) was associated with lower odds of smoking and drinking in female adolescents, while higher *p,p’*-DDE serum levels (aOR = 2.98, 95% CI 1.04–8.51, *p* = 0.04, per natural log unit increase) was associated with higher odds of the outcomes. Results of the mutually adjusted model were not significant for male adolescents. Further research to understand reasons for these sex-differences are warranted.

## 1. Introduction

Humans are exposed to a multitude of manmade chemicals that threaten health (Karlsson et al., 2020; Landrigan et al., 2018). The preponderance of epidemiological research investigates associations between exposures to single chemical contaminants and adverse health outcomes. However, this approach does not reflect real-life situations, as people are exposed to thousands of co-occurring, highly correlated chemicals (Wang et al., 2020). Because chemicals in mixtures can interact and may have greater, or lesser, than additive effects on biological processes, it is important to also study the combined chemical mixtures to better understand the role environmental contaminants play in human disease (Cassee et al., 1998; Karlsson et al., 2020; Kosnik et al., 2021; Vinggaard et al., 2021).

The adverse effects of contaminants not only depend on the dose and the chemical properties, but also the timing of the exposure during critical periods of development. Growing evidence demonstrates that exposures to even low contaminant levels during vulnerable time periods in utero and in early infancy can perturb development and lead to adverse outcomes (either frank disease or subtle symptoms) in childhood and/or later in life (Bianco-Miotto et al., 2017; Gluckman and Hanson, 2004; Rice and Barone, 2000). The placenta protects against some exposures, but depending on their lipophilicity, many chemical contaminants can reach the fetus through transplacental transfer (Vizcaino et al., 2014), and cross the blood-brain barrier, which is not fully developed until a few months after birth (Engelhardt and Liebner, 2014). Prenatal chemical exposure may for example interfere with dopaminergic activity and disrupt maternal and fetal thyroid hormone levels, both of which are essential for brain development, acting as a potential mechanism for childhood and adolescent behaviors (Factor--Litvak et al., 2014; Gibson et al., 2018; Whyatt et al., 2012).

Organochlorine pesticides (OCPs) were among the first pesticides produced and are classified as persistent organic pollutants (POPs) (Abubakar et al., 2020). With a primary source of ingestion of contaminated food (Schecter et al., 2010), these lipophilic chemicals can accumulate in adipose tissue, and be transferred across the placenta, posing a potential risk to the developing fetus (Herrera, 2002; Herrera and Ortega-Senovilla, 2010). The pesticide 1,1,1-trichloro-2,2-bis (p-chlorophenyl)ethane (*p,p’*-DDT), referred to generically as DDT, is likely the most well-known OCP. Although the production and usage of DDT were banned in the United States and most other developed countries in the 1970s, it is still extensively used in tropical developing countries to prevent the spread of malaria, dengue, leishmaniasis, and Japanese encephalitis by inhibiting mosquito growth (Abubakar et al., 2020). *p,p’*-DDT, its primary metabolite 1,1′-dichloro-2,2′-bis(p-chlorophenyl)ethylene (*p,p’*-DDE), and the isomer 1,1,1-trichloro-2-(p-chlorophenyl)-2-(o-chlorophenyl)-ethane (*o,p’*-DDT) that contaminates commercial DDT, are considered endocrine disrupting chemicals (EDCs), that may induce long-term effects on development and health after perinatal exposure (Alan S. Brown et al., 2018; Cohn et al., 2015; Wu et al., 2020). Studies have also demonstrated sex differences in the long-term response to perinatal DDT exposure in mice (La Merrill et al., 2014). Animal experiments further indicate that neurodevelopmental effects of early life DDT exposure are among the most sensitive outcomes (Eriksson et al. 1990, 1992; Johansson et al. 1995, 1996).

Although DDT was banned in the US in the 1970s, use of hexachlorobenzene (HCB) as a fungicide continued until 1984 and chlordane was in use to control termites until 1988. However, these chemicals, including the chlordane metabolite oxychlordane, remain detectable in soil (Harner et al., 1999), contaminated food (Schecter et al., 2010), and most of the US population (Control CfD, Prevention, 2019). Like DDT, lipophilic oxychlordane and HCB can store in adipose and be excreted in breastmilk (Bates et al., 1994; Savage et al., 1981). Animal models have shown associations between HCB and neurodevelopment, but results of epidemiological investigations have been conflicting (Korrick and Sagiv, 2008).

Research has suggested that developmental exposures may increase the risk of disruptive and disinhibitory behaviors in childhood and adolescence through impacts on neurodevelopment (Burns et al., 2013; Dickerson et al., 2019; Iacono et al., 2008; Rosenquist et al., 2017). Disinhibitory behaviors are defined as an assortment of conduct problems in adolescents, including disregard for authority, unsafe sexual behaviors, excessive use of illicit substances or other substances such as tobacco and alcohol. The onset of these risk behaviors shapes adult behavior and are associated with increased risk of poor educational attainment, future morbidity including substance use disorders and premature mortality (DuRant et al., 1999; Kipping et al., 2012; Olsson et al., 2016). To address the gap in research on the long-term influences of prenatal OCP exposure in adolescent risk behaviors, this prospective cohort study examines associations between prenatal exposure to a mixture of OCPs and their metabolites (i.e. *p,p’*-DDT, *p,p’*-DDE, *o, p’*-DDT, oxychlordane, and hexachlorobenzene (HCB)), both as single compounds and in combination, and the subsequent impact on the risk of smoking and alcohol use during adolescence in a sample randomly selected from the Child Health and Development Studies (CHDS) participants, based in Oakland and East Bay areas of California. We also investigate potential sex-differences in the response to prenatal OCP exposure.

## 2. Methods

### 2.1. Study design and participants

The CHDS was a prospective cohort study of prenatal determinants of offspring health in infancy, childhood, adolescence, and adulthood (van den Berg et al., 1988). In summary, pregnant mothers were recruited in the Oakland and East Bay areas of California between 1959 and 1967, peak years of DDT use in the United States (US) drawn from the Kaiser Permanente Health Plan (van den Berg et al., 1988). For mothers who agreed to participate in the CHDS (n = 20,754), mother-child pairs resulting in live births, excluding early adoptions and neonatal deaths, were followed through age 5 (van den Berg et al., 1988). Mother-child pairs who relocated outside of the Greater Bay area, were no longer members of the Kaiser Plan, or the child died before the age of 5 years were excluded at this stage, leaving 13,177 pairs for CHDS follow-up visits (van den Berg et al., 1988). There were 1752 children followed through adolescence, and for the purposes of this study, we used data from 600 randomly selected children whose mothers provided serum samples and had interview responses obtained from the mother during pregnancy and from the child between the ages of 15–17 years. This population has been previously described elsewhere (Keyes et al., 2011). Subjects from this subpopulation of the study must have completed the first two waves of the CHDS study at birth and from 9 to 11 years of age (van den Berg et al., 1988). To ensure independence of observations, the younger child of sibling pairs was removed from the analysis (n = 9). An additional 20 subjects were removed because no OCP exposure data were available. Subjects with no administered questionnaire (n = 10) or missing responses for smoking and drinking behaviors (n = 7) were also removed from the analysis, resulting in a sample of 554 adolescents (see Supplementary Fig. 1).

### 2.2. Exposure measurement

*In utero* exposure to OCPs was estimated using whole blood collected during the early postpartum period, within three days after delivery of the child between 1959 and 1967. Serum was isolated from the whole blood, aliquoted into 3 mL, and stored at − 20 °C until thawing for analysis in 2007–2008. Serum concentrations of OCPs and metabolites (*o,p*’-DDT, *p,p’*-DDT, *p,p’*-DDE, oxychlordane, and hexachlorobenzene (HCB)) were measured by mass spectrometry using previously described analytical methods (Brock et al., 1996; Gammon et al., 2002; Rogers et al., 2004; Sholtz et al., 2011; Wolff et al., 1993). Serum samples were analyzed in the laboratory of Dr. Mary Wolff in the Division of Environmental and Occupational Medicine at Mount Sinai School of Medicine (Brock et al., 1996; Gammon et al., 2002; Wolff et al. 1993, 1997). For regression analyses only lipid-adjusted values were used, with the exception of HCB which was not standardized for lipid analyses in the data provided. All OCP measures were also natural log-transformed for analysis. Limit of detection (LOD) was 0.80 ng/mL for individual OCPs based on three times the standard deviation of the levels found in the lowest quality control pool (Sholtz et al., 2011).

### 2.3. Measures of smoking and alcohol

Frequency, type, and amount of alcohol use along with smoking status were determined by responses to interviews administered by trained research staff to offspring at the age 15–17. Data collected on tobacco use included smoking status at the time of the interview and if the subject ever smoked at least one cigarette per day. Alcohol consumption was determined regarding the number of drinks per week, frequency of beer, wine, or other liquor consumption, common drinking partners (i.e. alone, with friends, with parents), and how often the subject was “high or tight”.

### 2.4. Statistical analysis

Highest education level between the two parents, derived based on the highest reported education level reported for each parent (high school graduate, trade school, some college, college graduate) and mother’s race (White, Black, other) were included as an *a* priori confounders based on previously reported associations with the exposures and outcomes of interest and was obtained from interview responses of the mother during pregnancy. Other demographic data, including mother’s and age during the pregnancy were abstracted from maternal and pediatric medical records and also included as *a priori* confounders. Additionally, we adjusted for the child’s age at the time of the interview.

The presence of drinking and smoking risk-taking behaviors was determined by responses to the interview questions indicating the child 1) Drank alcohol at least once per month (1 = yes, 0 = no) or 2) smoked tobacco products at the time of the questionnaire (1 = yes, 0 = no), resulting in a binary variable of no such behaviors (risk score = 0)vs. either or both of the behaviors (risk score >0). We used logistic regression analysis to obtain odds ratios (OR) and 95% confidence intervals (CI) for this analysis. To further investigate sex-differences in associations, these analyses were also stratified by reported sex at birth. This portion of the analysis was conducted using SAS, version 9.4.

We conducted the probit Bayesian Kernel Machine Regression (BKMR) to quantify the association of the mixture of OCPs with disinhibitory behaviors. This method allows to observe the non-linear association between exposure and outcome and to explore the potential interaction between exposures. The exposures included 5 OCPs or metabolites: *o,p’*-DDT, *p,p’*-DDT, *p,p’*-DDE, oxychlordane, and HCB. Exposure variables were right-skewed and therefore natural log-transformed. We standardized all exposure variables by their mean and standard deviation. Exposure outliers were defined as values smaller than −3 or greater than 3 on the standardized scale and were excluded from the analysis. We had two outcomes of interest, including a binary variable of having any drinking and smoking behaviors (risk score >0) and a binary variable of having drinking behaviors (drinking score >0). We adjusted for the previously mentioned confounders in BKMR analysis.

We used the “bkmr” R package to fit the probit BKMR with 1000 Markov chain Monte Carlo iterations. The model can be written as Y_i_* = *h*(Z_1i_, ..., Z_mi_) + μX_i_ + ε_i_, where *h* can be interpreted as the relationship between the exposures and some underlying, continuous latent variable Y*, which is a marker of a binary health outcome. We conducted variable selection and sampled from the posterior distribution of the subject-specific effects. Model convergence was evaluated visually using trace plots. Posterior inclusion probabilities (PIPs) were calculated for each exposure variable, which describes a measure of variable importance for each exposure to the model (Bobb et al., 2014). Dose-response functions for each exposure and outcome were plotted. We quantified the associations of individual exposure comparing 75th to 25th percentile at fixed levels of other exposures (25th, 50th, 75th percentiles). Overall association was evaluated by comparing the value of *h* (the relationship between the exposures and a continuous latent variable Y*), when all of the exposures are at a particular percentile to when all of them are at their median level. We also evaluated the two-way interaction visually by plotting the dose-response function for one exposure at the 25th, 50th, and 75th percentiles of another exposure.

## 3. Results

The majority of our study sample were White (70.9%). Within sex, there were no significant differences by race/ethnicity (see Table 1). The majority (59.5%) of our study sample was 17–18 years of age during the follow-up questionnaire. Regarding measures of socioeconomic status, 20.6% of the sample had a household income less than $5000 per year between 1959 and 1967, but there were no statistically significant differences between demographic distributions for males and females. Although current smoking status was comparable between males and females (13.9% vs 20.0%, *p* = 0.16, respectively), consumption of beer (p < 0.001) was higher in males compared to females, but consumption of and wine (*p* = 0.03) and mixed cocktails was higher in females (*p* = 0.002).

Table 2 shows the results of the analysis of alcohol and smoking behaviors adjusting for confounders. There were no significant associations found between pregnancy OCP and metabolite serum levels and this composite measure of behavior in adolescence. However, in the mutually adjusted logistic regression analysis, we observed a significantly increased risk of exhibiting monthly disinhibitory behaviors in subjects whose mothers had higher *p,p*’-DDE serum levels during pregnancy (OR) = 2.06, 95% CI 0.99–4.31, *p* = 0.05, per lipid adjusted natural log unit increase).

When we stratified analyses by sex at birth (Table 2), there were no statistically significant associations between OCPs and metabolites and smoking and drinking behaviors, though odds of were slightly lower for males and slightly higher for females with higher *o,p’*-DDT serum concentrations. In the mutually adjusted, sex-stratified analysis, risk of smoking and drinking behaviors was lower in adolescent females with higher maternal *o,p’*-DDT serum concentrations (OR = 0.26, 95% CI 0.09, 0.76, *p* = 0.01, per lipid-adjusted natural log unit increase) but elevated in association with higher maternal *p,p’*-DDE serum concentrations (OR = 2.98, 95% CI 1.04, 8.51 *p* = 0.04, per lipid-adjusted log unit increase). Results of the mutually adjusted model were not significant for male adolescents.

As shown in Supplemental Fig. 2, correlations between *p,p’*-DDE, *o, p’*-DDT, and *o,p’*-DDT were strong, ranging from 0.53 to 0.72. BKMR was used to assess overall associations between the OCP mixture and smoking and drinking behaviors in study participants with complete data (n = 534) when the compounds were designated as specific quantiles. PIPs for each OCP and metabolite are shown for each measure of behavior in Table 3. For the composite behavior measure, *o,p’*-DDT had the highest PIP (0.512), while for any alcohol consumption, HCB had the highest PIP (0.422). Although *o,p’*-DDT had the highest PIP in males when analyses of the composite smoking and drinking behaviors were stratified by sex (0.454), the highest PIP for this measure in females was seen for oxychlordane (0.366). Additionally, HCB had the highest PIP for females in sex-stratified analyses of any alcohol consumption (0.338), but PIPs for all OCPs in males were consistently low in males with highest seen for *p,p’*-DDE (0.266).

**Fig. 1.**
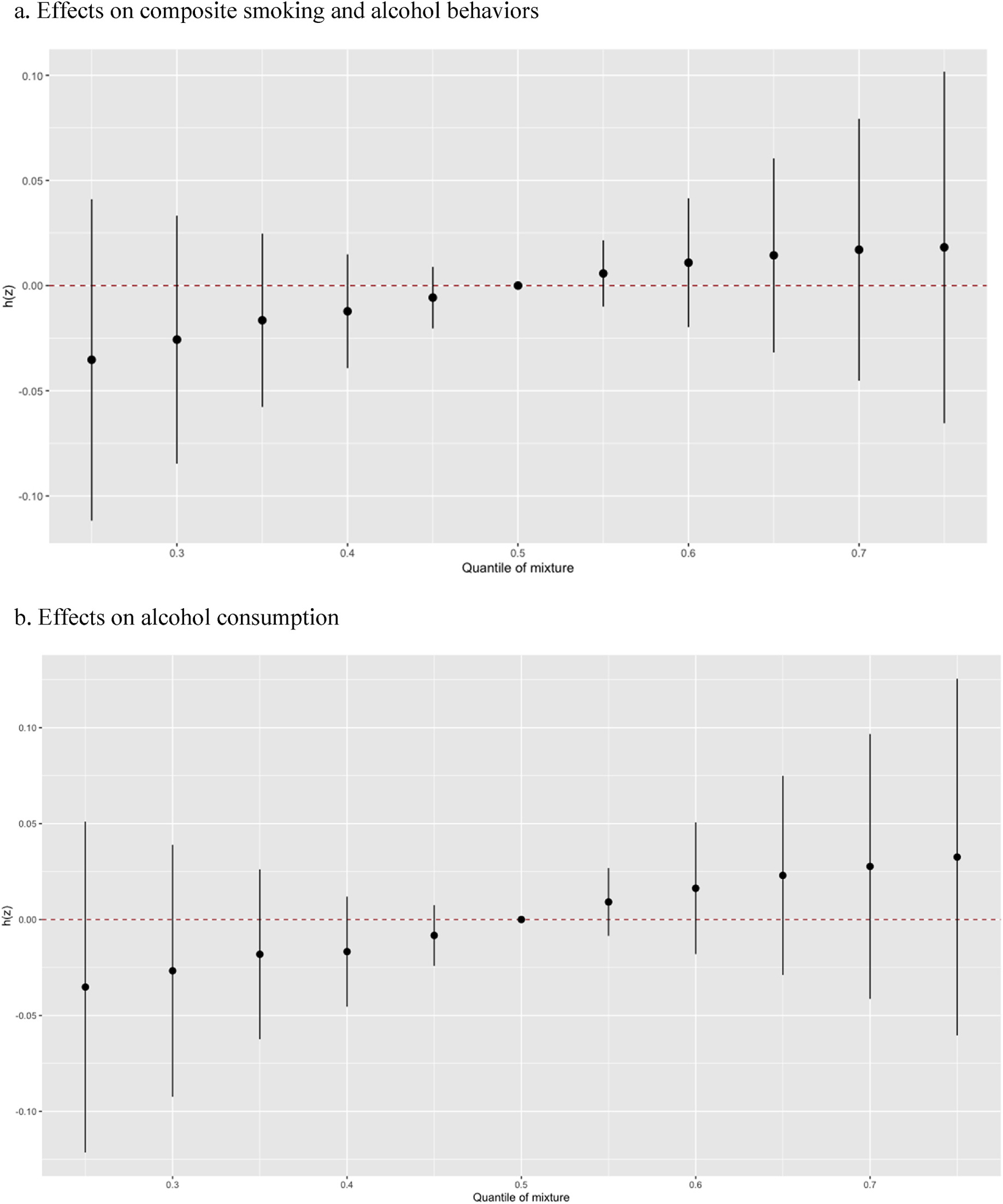
Overall effects of the mixture of six pregnancy serum OCPs on smoking and drinking behaviors in Bayesian kernel machine regression models.

**Fig. 2.**
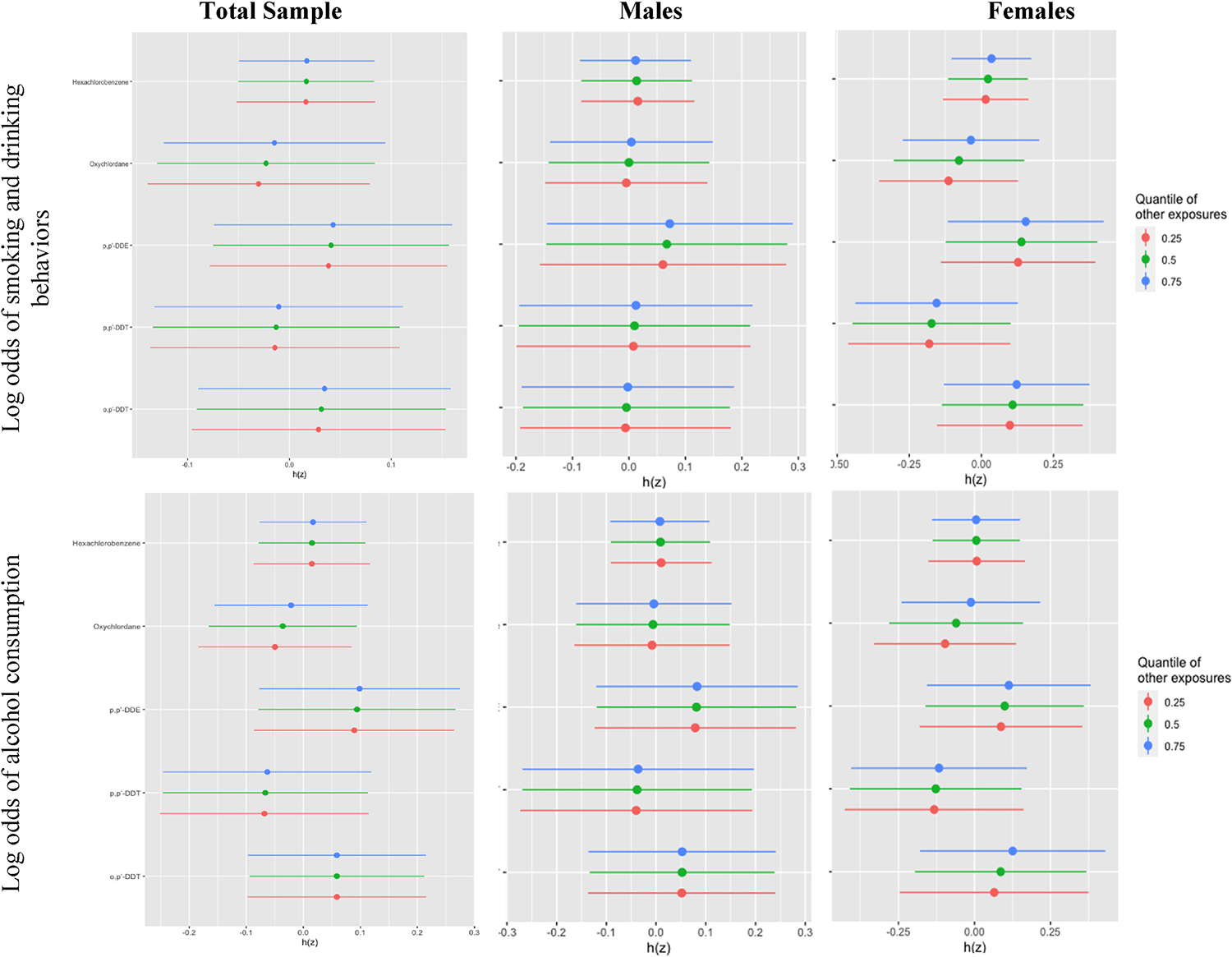
Single-exposure effects of each individual OCP on any smoking or drinking behaviors in Bayesian kernel machine regression.

**Fig. 3.**
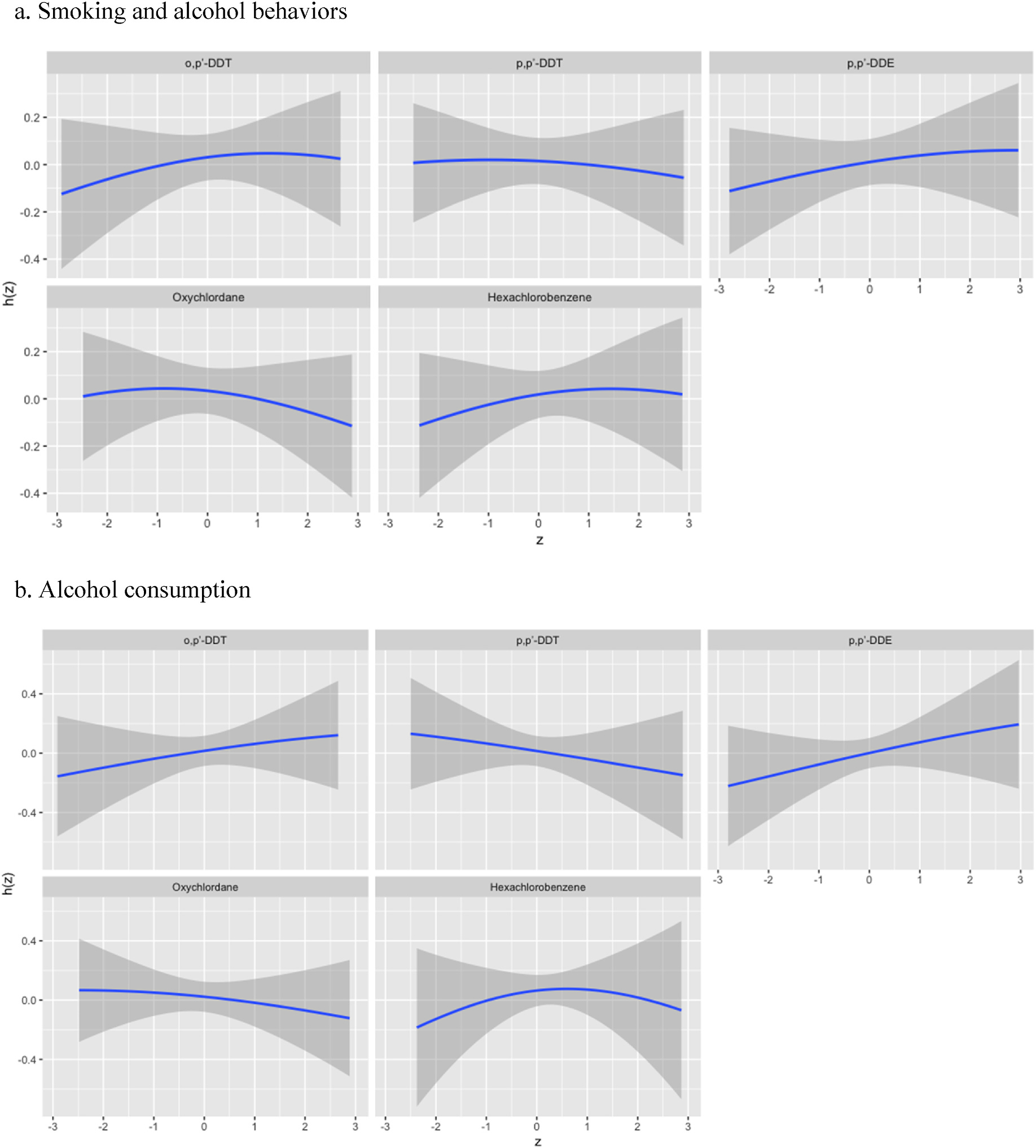
BKMR exposure-response function for smoking and drinking behaviors.

As shown in Fig. 1, although there was a trend for higher risk of behaviors with higher quantile of mixture, no statistically significant association was observed between the OCP mixture and any smoking and drinking behaviors. These results were consistent when results were stratified by sex at birth (not displayed). Fig. 2 displays single-exposure associations for each individual OCP and metabolite with any smoking and drinking behaviors when holding the other compounds as the 25th, 50th, and 75th percentiles. BKMR results did not identify a single OCP contributor to risk of smoking and drinking behaviors. Setting other OCPs at their median level, there was a slight increase in risk of smoking and drinking behaviors seen for oxychlordane from the 25th to 75th percentile, which was more prominent in females when results were stratified by sex. However, there does not appear to be a linear dose-response association for other mixture components for the combined sample, though there did appear to be a slight increase for *o,p’*-DDT in females. A positive nonlinear association was observed for *o,p’*-DDT and *p,p’*-DDE for both outcomes, although these associations were not statistically significant (Fig. 3), but no other association was observed for other OCPs and each outcome. These results were consistent for results for males in sex-stratified results, but a sharper negative linear association was observed for *p,p’*-DDT and combined smoking and drinking behaviors in females (Supplemental Fig. 3). Additionally, when evaluating the two-way interaction between OCPs (not displayed), there was no statistically significant interaction between OCP serum levels for either behavior measure.

## 4. Discussion

Today, we face a massive public health threat of increasing chemical exposures in the environment, which has documented effects on long-term health. Our prospective study is the first, to our knowledge, to examine whether prenatal exposure to OCPs is associated with alcohol and tobacco use in adolescent offspring. The results demonstrated that maternal *p,p’*-DDE serum levels were associated with increased smoking and alcohol use among adolescents. Additionally, we observed a significant increase in smoking and drinking behaviors in female adolescents of mothers with higher *p,p’*-DDE serum levels during pregnancy that was not seen in males. Although one study of maternal serum levels of DDE and HCB showed no association with offspring behavior from early childhood to adulthood in Denmark over 22 years of follow-up (Strøm et al., 2014), our results support a previous study of cord serum DDE levels that showed associations with childhood behavior outcomes like attention deficit hyperactivity disorder (ADHD) (Sagiv et al., 2010).

We observed several sex differences in our analysis. The observed sex differences could be due to toxicokinetic and toxicodynamic factors influenced by sex hormones and genes (Gochfeld 2007, 2017; Vahter et al., 2007), and is in line with an animal study demonstrating significant long-term effects of perinatal DDT exposure in female mice, but only minor effects in male mice (La Merrill et al., 2014). Although results from recent cohorts did not demonstrate significant differences between male and female adolescents (Cheng and Anthony, 2017; Slade et al., 2016), it is still possible that baseline differences in the prevalence of substance use between males and females may influence these associations. Specifically, other studies on sex differences in alcohol use have indicated higher alcohol consumption among men (Keyes et al., 2019). Similarly, our analysis and others using CHDS data showed significantly more drinking behaviors in boys in the 1970s compared to girls (Keyes et al., 2011), but research indicates that alcohol use among male adolescents is decreasing faster than use in female adolescents (Keyes et al., 2019; White, 2020). Likewise, observed smoking behaviors have historically been higher in girls (Keyes et al., 2011), but these trends continue to be apparent in recent examinations of contemporary tobacco use in adolescents (i.e. e-cigarettes) (Duan et al., 2021).

*In utero* exposure to *p,p’*-DDE could affect neurological development and offspring long-term behavior through several plausible biological pathways (Vrijheid et al., 2016). For example, it has been shown that human prenatal *p,p’*-DDE exposure may have life-long consequences through alterations of DNA methylation and gene expression (Wu et al., 2020; Yu et al., 2018). More specifically, *p,p’*-DDE may alter function and dopaminergic signaling in brain regions important for decision-making and pleasure-seeking behaviors (Bressan and Crippa, 2005; Koob, 2009; Seegal et al., 2005). Additionally, *in vitro* studies have revealed that *p,p’*-DDE also can impair regulation of dopamine homeostasis (Hatcher et al., 2008). Other possible mechanisms include impairment of early androgen influences on neural and behavioral development, as *p,p*′-DDE can inhibit androgen receptor binding, androgen-induced transcriptional activity, and androgen action (Hines, 2008; Kelce et al., 1995; McCarthy, 2008). However, more studies are needed to further elucidate mechanisms by which *p,p’*-DDE may disrupt neurodevelopment.

When evaluating prenatal co-exposure to the measured OCPs with BKMR, we noticed a trend in higher risk of smoking or alcohol use among adolescents with higher quartile mixture levels, though these results were not significant. There also appeared to be a dose-response association with oxychlordane, but there was no interaction between OCPs and none of these associations were statistically significant. These results are consistent with our previous analysis of another set of POPs, which showed few associations between PCBs and these behaviors (Dickerson et al., 2019). Therefore, our findings for maternal POP mixtures and offspring adolescent behaviors are inconclusive, but highlight the importance of considering such mixtures of environmental toxicants in environmental epidemiologic investigations, and to further develop statistical approaches to estimate the effects of multiple exposures (Stafoggia et al., 2017).

It is important to note that although our study was strengthened by using prospectively collected exposure measures, our study also has some limitations. As adolescent behaviors were based on self-report to interviewers, there may be some inherent measurement error in our outcomes of interest. However, under the assumption that any reporting differences would not differ by OCP maternal exposures, this potential misclassification may have biased our results towards the null by weakening the observed exposure-outcome association. We also recognize that in using lipid standardization for our OCP measures, via division of serum concentrations by serum lipids, we may increase the risk of bias, though we would expect this to be minimal (Schisterman et al., 2005). Furthermore, OCPs are lipophilic and can thus store in fat and be transferred to breastfed infants; however, we had no information on breastfeeding to evaluate the impact of this potential window of exposure. Considering the outcome of interest for our analysis is during adolescence, we do not anticipate that this potential route of infant exposure would greatly impact results. Additionally, indirectly adjusting for highest parental education level, we may have indirectly adjusted for breastfeeding, which was associated with SES during the period of data collection (1959–1967) (Lactation IoMUCiNSDPa, 1991). Likewise, because study subjects reached adolescence during the 1970s and trends in alcohol, cigarette, and other substance use have dramatically reduced since this time(Johnson and Gerstein, 1998), our results may not be generalizable to current trends in adolescents, though they may lend themselves to hypotheses in risks factors for other more popular risk-taking behaviors such as vaping. Furthermore, although DDT and DDE were also banned in the US in 1972, they continue to serve as a source of concern as they persist in the environment for several decades and still are extensively used in developing countries (IARC, 2018).

## 5. Conclusion

It is important to identify the factors that influence the onset of smoking and drinking behaviors in adolescence, which is a strong predictor of continued use and abuse into adulthood (DuRant et al., 1999; Kipping et al., 2012; Olsson et al., 2016). In line with the recognized adverse impact of OCP exposures on child neurodevelopment (Grandjean and Landrigan, 2014), our results provide support for an increased risk of drinking and smoking behaviors in individuals whose mothers had higher *p,p’*-DDE serum levels during pregnancy. Our findings also provide evidence that risk of adolescent smoking and drinking behaviors due to prenatal environmental exposures may be modified by sex and emphasize the importance of these exposures to women of childbearing age. It is warranted to conduct additional research on this topic, replicating our study in different and larger longitudinal cohorts, and perform detailed experimental studies of potential underlying mechanisms.

## Supplementary Material

Appendix A. Supplementary Data

## Figures and Tables

**Table 1:** Descriptive statistics for study subjects (n = 554).

		Male (n = 274)		Female (n = 280)		
			
		N	%	N	%	*p*

Age (Years)	15–16	108	39.41	116	41.43	0.677
	17–18	166	60.59	164	58.57	
Mother’s age at child’s birth (years)	15–24	66	24.09	86	30.71	0.188
	25–34	154	56.20	139	49.64	
	35–47	54	19.71	55	19.64	
Mother’ s race	White	200	72.99	193	68.93	0.524
	Black	53	19.34	57	20.36	
	Hispanic	9	3.28	9	3.21	
	Asian	12	4.38	20	7.14	
	Other	0	0	1	0.36	
Mother’s Education	Less than high school	31	11.31	33	11.79	0.447
	High school graduate	82	29.93	98	35.00	
	Trade school	17	6.20	18	6.43	
	Some college	86	31.39	68	24.29	
	College graduate	58	21.17	62	22.14	
	Missing	0	0	1	0.36	
Father’s Education	Less than high school	37	13.50	44	15.71	0.140
	High school graduate	83	30.29	84	30.00	
	Trade school	14	5.11	6	2.14	
	Some college	66	24.09	54	19.29	
	College graduate	73	26.64	91	32.50	
	Missing	1	0.36	1	0.36	
Household Income	<$5000	56	20.44	58	20.71	0.914
	$5000 to $10,000	152	55.47	155	55.36	
	>$10,000	37	13.50	42	15.00	
	Missing	29	10.58	25	8.93	
Adolescent Current Smoking (yes/no)		38	13.87	56	20.00	0.155
Adolescent Alcohol Consumption (ever/never)	Beer ^a^	167	64.23	112	42.26	**<0.001**
	Wine ^b^	94	36.43	121	46.18	**0.027**
	Mixed cocktails ^c^	106	75.85	210	76.92	**0.002**

*p*-value based on chi-square test.

a Beer consumption missing for 14 (5.10%) males and 15 (5.36%) females.

b Wine consumption missing for 16 (5.84%) males and 18 (6.43%) females.

c Mixed cocktails consumption missing for 9 (3.28%) males and 7 (2.50%) females.

**Table 2:** Odds of drinking and smoking behaviors.

Serum OCP (ng/mL)	Mean ± SD	Median (IQR)	Adjusted OR (95% CI)	*P* - value	Mutually Adjusted OR (95% CI)	*P* - value

Total Sample (n = 517)						
*o,p’*-DDT	0.58 ± 0.55	0.43 (0.28–0.68)	0.98 (0.76, 1.27)	0.85	0.95 (0.64, 1.41)	0.80
*p,p’*-DDT	12.89 ± 7.71	10.88 (8.04–15.14)	0.89 (0.60, 1.32)	0.57	0.63 (0.30, 1.29)	0.21
*p,p’*-DDE	44.48 ± 23.46	39.23 (30.14–53.19)	1.19 (0.78, 1.80)	0.43	**2.06 (0.99, 4.31)**	**0.05**
Oxychlordane	0.58 ± 0.36	0.47 (0.37–0.67)	0.91 (0.64, 1.28)	0.58	0.83 (0.53, 1.32)	0.44
Hexachlorobenzene ^a^	0.40 ± 0.43	0.34 (0.24–0.45)	1.12 (0.96, 1.31)	0.16	1.11 (0.94, 1.31)	0.22
Males (n = 256)						
*o,p’*-DDT	0.56 ± 0.52	0.41 (0.28–0.63)	0.84 (0.55, 1.29)	0.43	0.62 (0.32, 1.19)	0.15
*p,p’*-DDT	12.50 ± 7.41	10.96 (8.11–14.50)	1.10 (0.64, 1.46)	0.76	1.30 (0.46, 3.66)	0.62
*p,p’*-DDE	41.99 ± 20.06	37.43 (29.99–50.09)	1.34 (0.69, 2.62)	0.39	2.01 (0.66, 6.13)	0.22
Oxychlordane	0.51 ± 0.32	0.43 (0.35–0.56)	1.03 (0.56, 1.89)	0.92	0.73 (0.31, 1.69)	0.46
Hexachlorobenzene ^a^	0.42 ± 0.48	0.34 (0.26–0.45)	0.94 (0.60, 1.46)	0.77	0.93 (0.59, 1.47)	0.76
Females (n = 261)						
*o,p’*-DDT	0.61 ± 0.59	0.44 (0.28–0.76)	1.09 (0.78, 1.53)	0.62	1.31 (0.78, 2.20)	0.30
*p,p’*-DDT	10.80 ± 7.99	10.80 (8.04–16.07)	0.73 (0.42, 1.25)	0.25	**0.26 (0.09, 0.76)**	**0.01**
*p,p’*-DDE	42.27 ± 26.16	42.27 (30.15–56.70)	1.16 (0.66, 2.01)	0.61	**2.98 (1.04, 8.51)**	**0.04**
Oxychlordane	0.65 ± 0.39	0.53 (0.40–0.77)	0.91 (0.58, 1.41)	0.66	0.92 (0.51, 1.64)	0.77
Hexachlorobenzene ^a^	0.38 ± 0.37	0.33 (0.20–0.46)	1.12 (0.94, 1.34)	0.22	1.11 (0.92, 1.34)	0.27

All models adjusted for child’s age at the time of the interview, mother’s race, mother’s age at time of birth, and highest parental education with lipid-adjusted OCP concentrations.

Mutually adjusted models simultaneously include all serum OCP measures + previously adjusted confounders.

IQR = 25th percentile to 75th percentile.

Missing values: *o,p’*-DDT = 7, *p,p’*-DDT = 2, *p,p’*-DDE = 3, Oxychlordane = 4, Hexachlorobenzene = 9.

a Hexachlorobenzene measures were not lipid adjusted for regression analyses.

**Table 3:** OC pesticide posterior inclusion probabilities.

	Any Smoking or Drinking Behaviors	Any Alcohol Consumption

Total sample		
*o,p’*-DDT	0.512	0.280
*p,p’*-DDT	0.362	0.238
*p,p’*-DDE	0.462	0.244
Oxychlordane	0.450	0.270
Hexachlorobenzene	0.494	0.422
Male		
*o,p’*-DDT	0.454	0.282
*p,p’*-DDT	0.248	0.224
*p,p’*-DDE	0.452	0.266
Oxychlordane	0.330	0.206
Hexachlorobenzene	0.414	0.252
Female		
*o,p’*-DDT	0.236	0.164
*p,p’*-DDT	0.306	0.168
*p,p’*-DDE	0.266	0.160
Oxychlordane	0.366	0.218
Hexachlorobenzene	0.246	0.388

## Abbreviations:

OCPs: Organochlorine pesticides
POPs: persistent organic pollutants
*p,p’*-DDT: 1,1,1-trichloro-2,2-bis(p-chlorophenyl)ethane ()
*p,p’*-DDE: 1,1′-dichloro-2,2′-bis(p-chlorophenyl)ethylene
*o,p’*-DDT: 1,1,1-trichloro-2-(p-chlorophenyl)-2-(o-chlorophenyl)-ethane
EDCs: endocrine disrupting chemicals
HCB: hexachlorobenzene
PIPs: Posterior inclusion probabilities
BKMR: Bayesian Kernel Machine Regression
