# Supplementary material for: Associations of prenatal exposure to mixtures of organochlorine pesticides and smoking and drinking behaviors in adolescence
Source: Environ Res. Author manuscript; Available in PMC 2024 May 21. (PMC11108254; doi:10.1016/j.envres.2021.112431)
Supplement: Appendix A. Supplementary Data [file NIHMS1990835-supplement-Appendix_A__Supplementary_Data.docx]

**Supplemental Figure 1. Study inclusion**

Pregnant mothers consented for CHDS

(n=20,754)

Mother-child pairs excluded

(n = 2172)

Fetal deaths = 1710

Neonatal deaths = 293

Early adoptions = 169

Children observed at infancy

(n = 18,582)

Children excluded

(n = 5405)

Lost to follow-up = 1411

Died = 126

Relocated = 1445

No longer insured by Kaiser = 2423

Children observed at 5 years old

(n = 13,177)

Relocated, lost to follow-up, refused participation (n = 9440)

Children included in 9-11 study

(n = 3737)

Relocated, lost to follow-up, refused participation (n = 9440)

Children participated in Adolescent Study at 15-17 years

(n = 2020)

Did not participate in behavior interview (n = 268)

Children interviewed at age 15-17 years

(n = 1752)

Children included for analyses

(n = 554)

Children excluded

(n = 46)

Younger sibling = 9

No OCP exposure data = 20

No behavior questionnaire = 10

No alcohol and smoking responses = 7

Children randomly sampled for study

(n = 600)

**Supplemental Figure 2. Heat map of correlations between OCPs**


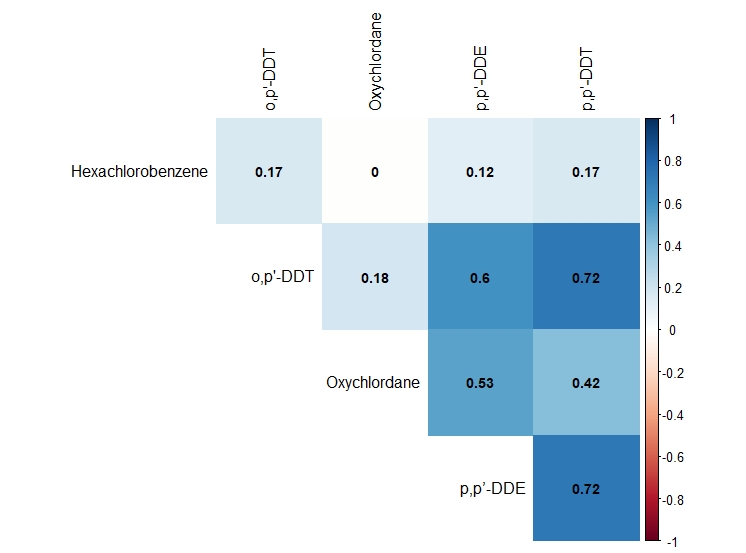


**Supplemental Figure 3. BKMR exposure-response function for smoking and drinking behaviors stratified by sex.**

S1.a. Smoking and alcohol behaviors in males


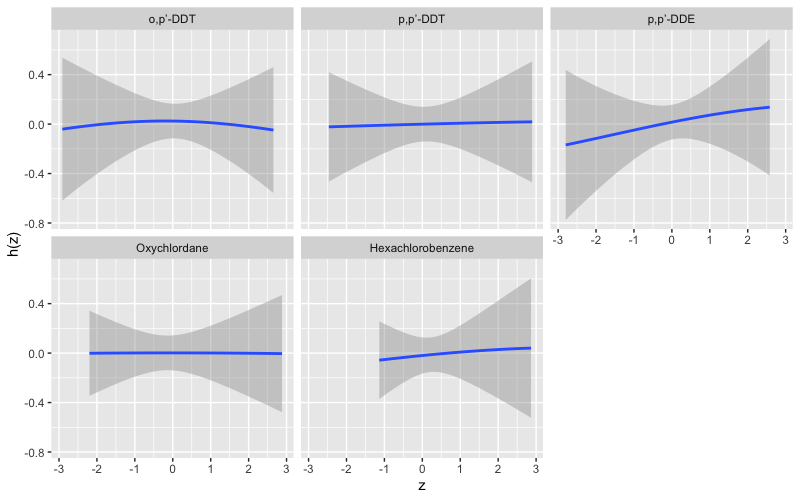


S1.b. Smoking and alcohol behaviors in females
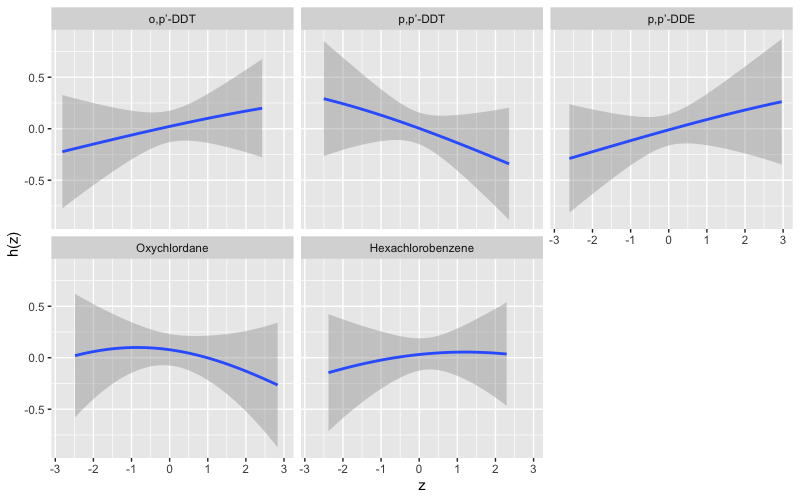


S1.c. Alcohol consumption in males


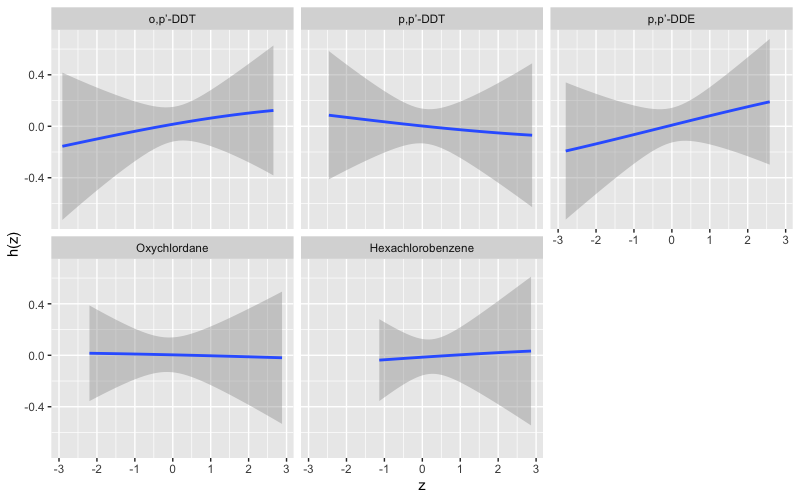


S1.d. Alcohol consumption in females


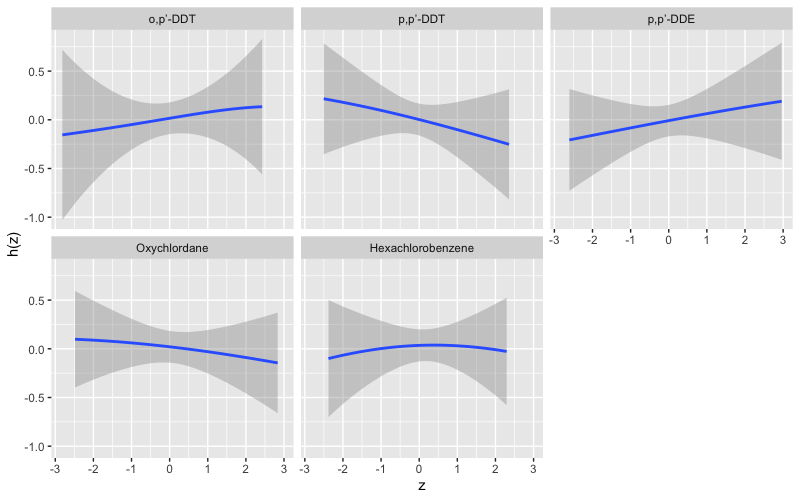


All models adjusted for child’s age at the time of the interview mother's race, mother's age at time of birth, and highest parental education
